# DISORGANIZATION, FEAR AND ATTACHMENT: WORKING TOWARDS CLARIFICATION

**DOI:** 10.1002/imhj.21689

**Published:** 2018-01-04

**Authors:** Robbie Duschinsky

**Affiliations:** ^1^ University of Cambridge

**Keywords:** attachment, disorganization, fear, rejection, dysregulation, afectividad, desorganización, miedo, rechazo, desregulación, Attachement, Désorganisation, Peur, Réjection, Dysrégulation, Bindung, Desorganisation, Angst, Ablehnung, Dysregulation, 愛着, 無秩序, おそれ, 拒絶, 調節不全, 依附, 混亂, 恐懼, 排斥, 失調, التعلق, الاضطراب, الخوف, الرفض‐ الخلل في التنظيم

## Abstract

In 1990, M. Main and J. Solomon introduced the procedures for coding a new “disorganized” infant attachment classification for the Ainsworth Strange Situation procedure (M.D.S. Ainsworth, M. Blehar, E. Waters, & S. Wall, 1978). This classification has received a high degree of interest, both from researchers and from child welfare and clinical practitioners. Disorganized attachment has primarily been understood through the lens of E. Hesse and M. Main's concept of “fright without solution,” taken to mean that an infant experiences a conflict between a desire to approach and flee from a frightening parent when confronted by the Strange Situation. Yet, looking back, it can be observed that the way Hesse and Main's texts were formulated and read has generated confusion; there have been repeated calls in recent years for renewed theory and clarification about the relationship between disorganization and fear. Responding to these calls, this article revisits the texts that introduced the idea of fright without solution, clarifying their claims through articulating more precisely the different meanings of the term *fear*. This clarified account will then be applied to consideration of pathways to infant disorganized behaviors.

## RESUMEN

En 1990, Main y Solomon introdujeron procedimientos para codificar una nueva clasificación de la afectividad ‘desorganizada’ del infante para el procedimiento de la Situación Extraña de Ainsworth. Esta clasificación ha recibido un alto grado de interés, tanto por parte de investigadores como de profesionales de bienestar infantil y clínico. La afectividad desorganizada ha sido primariamente comprendida a través del lente del concepto ‘susto sin solución’ de Hesse y Main, cuyo significado se entiende como el hecho de que un infante a quien se le confronta con la Situación Extraña experimenta un conflicto entre un deseo de acercamiento y de escaparse de un progenitor que le asusta. Retrospectivamente, se puede observar que la manera como los textos de Hesse y Main fueron formulados y leídos ha generado confusión; se han dado repetidas llamadas en años recientes clamando por la renovación teórica y aclaración acerca de la relación entre desorganización y miedo. Como respuesta a esa llamada, este ensayo vuelve a tratar los textos que introdujeron la idea de ‘susto sin solución,’ aclarando sus afirmaciones por medio de articular con más precisión los diferentes significados del término ‘miedo.’ Esta versión tal como se vuelve a especificar será entonces aplicada a la consideración de trayectorias para el comportamiento desorganizado de los infantes.

## RÉSUMÉ

En 1990, Main and Solomon ont introduit les procédures pour le codage d'une nouvelle classification d'attachement « désorganisé » du nourrisson pour la procédure de Situation Etrange d'Ainsworth Strange Situation procédure. Cette classification a fait l'objet d'un grand intérêt, à la fois de la part des chercheurs et de la part des professionnels de la protection sociale et cliniques. L'attachement désorganisé a avant tout été compris à travers le prisme du concept de Hesse et Main de « peur sans solution », interprété comme voulant dire qu'un nourrisson fait l'expérience d'un conflit entre un désir d'approcher et de fuir un parent effrayant lorsqu'il est confronté par la Situation Etrange. Cependant si l'on regarde en arrière on peut observer que la manière dont les textes de Hesse et de Main ont été formulés et lus a généré pas mal de confusion et dans les années récentes on a vu bien des appels répétés pour une théorie renouvelée et une clarification de la relation entre la désorganisation et la peur. Cet article répond à ces appels et réexamine les textes qui ont présenté l'idée de « peur sans solution », clarifiant leurs argumentations en articulant plus précisément les différentes significations du terme de « peur ». Ce compte‐rendu re‐spécifié sera alors appliqué dans considération de trajectoires de comportements désorganisé du nourrisson.

## ZUSAMMENFASSUNG

Im Jahr 1990 führten Main und Solomon die Verfahren zur Kodierung einer neuen Klassifikation der “desorganisierten” Bindung des Säuglings für den Fremde‐Situations‐Test von Ainsworth ein. Diese Klassifikation stieß auf großes Interesse, sowohl bei den Forschern als auch bei der Kinderfürsorge und der klinischen Praxis. Desorganisierte Bindung wurde in erster Linie anhand des Hesse und Main Konzeptes von “Schrecken ohne Lösung” verstanden. Das heißt, dass ein Säugling einen Konflikt zwischen dem Wunsch, sich einem beängstigenden Elternteil zu nähern und vor ihm zu fliehen, erlebt, wenn er mit der fremden Situation konfrontiert wird. Rückblickend lässt sich jedoch feststellen, dass die Art und Weise, wie die Texte von Hesse und Main formuliert und gelesen wurden, für Verwirrung gesorgt hat. In den letzten Jahren wurden immer wieder Forderungen nach einer neuen Theorie und Klärung des Verhältnisses zwischen Desorganisation und Angst laut. Als Reaktion auf diese Aufrufe werden in diesem Artikel die Texte, die die Idee des “Schreckens ohne Lösung” eingeführt haben, erneut aufgegriffen und ihre Behauptungen klarer formuliert, indem die verschiedenen Bedeutungen des Begriffs “Angst” präzisiert werden. Diese neu spezifizierte Beschreibung wird dann im Hinblick auf die Betrachtung von Wegen hin zu desorganisierten Verhaltensweisen von Säuglingen angewendet.

## 抄録

**「解決法のない恐怖Fright without Solution」を再規定する: 乳児の無秩序型愛着、恐怖、および調整**

1990年に、MainとSolomonは、 AinsworthのStrange Situation法に新しい「無秩序型disorganized」乳児愛着分類のコード化手順を導入した。この分類は研究者、そして子どもの福祉と臨床家の両者から、とても強い興味を集めてきた。無秩序型の愛着は、HesseとMainの概念である「解決法のない恐怖fright without solution」のレンズを通して主として理解されてきた。それは、Strange Situation法によって直面させられた時に、恐ろしい親に近づきたい気持ちと逃げたい気持ちの間の葛藤を体験している乳児を意味していた。しかしふりかえると、HesseとMainの文章が規定され読まれた方法が、混乱を引き起こしてきたことが観察された;近年、無秩序と恐怖の間の関係性について、理論の更新と明確化への要求が繰り返されてきた。これらの要求に応えて、この論文では「解決法のない恐怖」の考えを紹介した文章を再訪し、「恐怖fear」の様々な意味をより厳密に述べることを通して、彼らの主張を明確化する。この再規定した説明は、次に乳児の無秩序な行動への道筋を考察することに適応されるだろう。

## 摘要

重新設定'沒有解決方案'的恐懼:嬰兒混亂型依附丶恐懼和調節

1990年, Main和Solomon介紹了編碼Ainsworth陌生情境一項新的“混亂”嬰兒依附分類程序。這分類方法受到研究人員丶兒童福利機構和臨床醫生的高度關注。混亂型依附主要是通過Hesse和Main “沒有解決的恐懼” 的概念來理解, 意思是說, 嬰兒在遇到陌生的情況時, 想要接近和逃離受驚的父母。然而, 回顧一下, 可以看出, Hesse和Main文本被制定和閱讀的方式引起了混亂; 近年來學者一再呼籲, 重新設定理論和澄清混亂和恐懼之間的關係。為了回應這些要求, 本文重新提出引入“沒有解決之道”這個概念的文本, 通過更準確地闡述“恐懼”這個術語的不同含義, 來澄清他們的主張。這個新的說法將用來理解嬰兒混亂行為。

## ملخص

**إعادة تحديد مفهوم “الخوف بدون حل”: التعلق المضطرب عند الرضع والخوف والتنظيم **

في عام 1990 قدم (ماين وسولومون) إطار اجراءات لترميز نوع جديد من اضطرابات التعلق ضمن تصنيف آينسوورث لاجراءات الموقف الغريب. وقد حظي هذا التصنيف بدرجة عالية من الاهتمام من الباحثين ومن الممارسين في مجال رعاية الطفل. وتم تناول وفهم التعلق الغير المنظم في ضوء مفهوم (هيسي وماين) عن “الخوف بدون حل” والذي يشير إلى إحساس الطفل بصراع بين رغبة في الاقتراب ودافع الابتعاد عن الوالد المخيف عند مواجهة سيناريو الموقف الغريب. ومن جانب آخر يمكن ملاحظة أن طريقة صياغة (هيسي وماين) للمفهوم ربما يشوبها بعض الغموض. فقد كانت هناك دائما دعوات متكررة في السنوات الأخيرة لتجديد النظرية وتوضيح العلاقة بين اضطراب التعلق والخوف. هذا البحث يعيد النظر في ما كتب عن فكرة “الخوف بدون حل” مع توضيح المفاهيم الأساسية من خلال تحديد المعاني المختلفة لمصطلح “الخوف”. هذا النهج المحدد سوف يتم تطبيقه في الرؤى المختلفة التي تتناول مسارات سلوكيات الأطفال المضطربة وغير المنظمة.

Bowlby (1969) argued that on perceiving threat or discomfort, primate infants will be disposed to seek the physical and attentional availability of their familiar caregiver through signals and movements, including crying, smiling, and crawling. He termed this disposition the *attachment system*, and described caregiver physical and attentional availability as the “set‐goal” of the attachment system in infancy. The Ainsworth Strange Situation elicits individual differences through activating the attachment system. A caregiver leaves the infant twice in a novel environment with interesting toys, first with a stranger and then alone, before returning (Ainsworth, Blehar, Waters, & Wall, 1978). Three patterns of behavior were initially identified by Ainsworth Blehar, Waters, and Wall (1978). When the attachment system is activated in the Strange Situation, infants who anticipate that their caregiver will be responsive seek proximity with the caregiver directly and are soothed by this contact. In doing so, these secure (B) infants can satisfy the demands of the attachment system for caregiver availability directly and then can return to exploration. Avoidant (A) infants direct their attention away from the demands of the attachment system, masking their distress through a focus on the toys. Avoidance was theorized as an attempt by the infant to indirectly achieve what availability that they can from a caregiver who they expect would rebuff them if they showed distress. Ambivalent/resistant (C) infants likewise alter the output of the attachment system in the service of maximizing the availability of their caregiver; however, these infants utilize a preoccupying attention to the caregiver, and displays of anger (C1) and/or passive, helpless distress (C2), to maintain the attentiveness of a caregiver who they anticipate might otherwise be only erratically responsive. Although the infants seek the availability of the caregiver in the Strange Situation, those classified as ambivalent/resistant are not soothed by reunion or able to return to play.

A fourth Strange Situation classification, “disorganized/disoriented,” was added by Main and Solomon (1986, 1990). It emerged out of observations of infant behaviors discrepant with the Ainsworth classifications. Infant behaviors coded as disorganized/disoriented were clustered based on their apparent morphology into seven indices:

- Sequential displays of contradictory behavior;
- Simultaneous display of contradictory behavior;
- Undirected, misdirected, or incomplete movements;
- Stereotypies, mistimed movements, and anomalous postures;
- Freezing or stilling;
- Display of apprehension of the caregiver; and
- Overt signs of disorientation or disorganization.

The term *disorganization* was used by Main and Solomon (1986, 1990) to refer to both (a) conflict at the level of the attachment system, undermining the coherence of its behavioral expression; and (b) the behaviors that suggest such disruption. It also was then used to (c) refer to the classification (Duschinsky & Solomon, 2017). In reflecting on the possible cause of these behaviors, Main and Hesse (1990) proposed that they could be expected to result if the infant were to experience the attachment figure as a stimulus eliciting alarm. According to Bowlby (1960, 1969), the attachment system impels an infant to seek proximity to his or her familiar caregiver when anxious, even if that caregiver is punitive or otherwise alarming. So Main and Hesse (1992 1992) hypothesized that alarm associated with the attachment figure him‐ or herself would lead to a biologically channeled, paradoxical demand to both approach and take flight from the caregiver: “The attached infant is *biologically* rather than externally driven to perceive/respond to this single element of the environment [the caregiver] in completely opposing ways. Moreover its flight and approach tendencies, both vital to survival, are mutually exacerbating” (p. 338; also see Solomon & George, 1999a).

In an article from 1999, intended to supersede earlier statements of their hypothesis, Hesse and Main emphasized that a paradox between a desire to approach and flee from the parent can be prompted for infants of parents who are in no way physically maltreating. These authors drew on interview data with the caregivers of infants classified as disorganized/disoriented, which found evidence of a lack of resolution for trauma or loss. “As a result of their own traumatic experiences or frightening ideation,” it was suggested that these caregivers may alarm their infant “via the exhibition of frightened, dissociated, or anomalous forms of threatening behavior” (Hesse & Main, 1999, p. 483). They argued that to the degree that an infant was unable to find a safe haven in the caregiver due to this conflict, the infant could be anticipated to be faced with a predicament of “fright without solution” (Hesse & Main, 1999, p. 484). This process was hypothesized as a pathway to disorganized/disoriented infant attachment behavior, as seen in the Strange Situation.

From 1992, Hesse and Main began to formulate a coding system for the kinds of behaviors that may lead to fright without solution. This “FR” system coded three kinds of parental behavior that are assumed to alarm the infant. The first is directly threatening or predatory behaviors, such as barred teeth. A second is dissociative displays, which are assumed to frighten the infant through the caregiver's inexplicable attentional unavailability. The third is displays by the caregiver of fear of the infant themselves, which are likewise thought to make the infant afraid and unable to seek a safe haven in the caregiver: “Danger that originates from within the self is intrinsically inescapable” (Hesse & Main, 2006, p. 323). Hesse and Main (2006) later added three further kinds of behavior that are not themselves assumed to lead to an approach–flight paradox but which suggest an altered state of consciousness expected to be indirectedly linked to this outcome: (a) caregiver behaviors treating the infant as if he or she was an attachment figure, behaving in a timid or deferential way toward the child (also see Lyons‐Ruth et al., 2013); (b) sexual behaviors toward the infant by the caregiver, which suggest confusion between the caregiving and sexual systems as well as a lack of capacity to monitor action; and (c) behaviors by the parent that are coded in the Main and Solomon (1990) indices for infant disorganization/disorientation, such as briefly moving in a stiff, asymmetrical, robot‐like manner.

Empirical evidence has supported the predicted association between infant disorganized/disoriented attachment in the Strange Situation and caregiver behaviors likely to alarm an infant, coded with the FR coding system. Madigan et al. (2006) reported from a meta‐analysis that infants whose parents displayed frightened, frightening, or dissociative behavior were 3.7 times more likely to be classified as “disorganized/disoriented” in the Strange Situation (*r* = .34, *N* = 851; also see Jacobvitz, Hazan, Zackagnino, Mesina, & Beverung, 2011). In addition, longitudinal studies have demonstrated links between infant disorganized attachment and themes of fear in doll‐play and family drawings in middle‐childhood (e.g., Fury, Carlson, & Sroufe, 1997). The classification has been found to predict adverse behavioral sequelae such as externalizing conditions (Fearon, Bakermans‐Kranenburg, van IJzendoorn, Lapsley, & Roisman, 2010).

Although the Main and Hesse hypothesis has been influential, sometimes this influence has not been in line with the hopes of the authors. In particular, there has been “fuzziness” in how the concepts of fear and disorganization have been interpreted. In terms of policy and practice, the Main and Hesse hypothesis has been read by many as suggesting that infants who show disorganized attachment behaviors must be *afraid of* their caregiver. Granqvist et al. (2016) recently documented how misunderstanding of the relationship between disorganization and fear has led social workers to assume that any infant who shows behavior in the Main and Solomon (1990) indices will have experienced inadequate experiences of parenting and require the attention of social services.

Also within the research community, there have been calls for renewed theory and clarification around this topic. One set of calls has come from clinically oriented researchers who have expressed particular interest in pathways to attachment disorganization in a child's relationship with his or her caregiver, and the role of different kinds and qualities of fearful experience (e.g., Beebe & Lachmann, 2014; Slade, 2014; Zeanah & Gleason, 2010). A second set of calls has come from attachment researchers in the social psychology tradition, who have developed a “fearful attachment style.” This was modeled on disorganized attachment, but it certainly remains unclear whether or how the two constructs relate to one another, conceptually or empirically (Mikulincer & Shaver, 2016; Paetzold, Rholes, & Kohn, 2015). Finally, a third set of calls has come from researchers from the developmental tradition of attachment research, who, while acknowledging that the disorganized classification addresses phenomena of clinical relevance, have interests in potential differences among these behaviors (e.g., Crittenden, 1999; Padrón, Carlson, & Sroufe, 2014; Waters & Crowell, 1999). Most recently, Lyons‐Ruth and Jacobvitz (2016) urged researchers to examine which among the forms of disorganization especially predict later risk.

It is not the goal of this article to address these varied literatures. However, these calls highlight the need for greater conceptual clarity regarding the fear–disorganization relationship. This article will begin by clarifying the Main and Hesse hypothesis, acknowledging imprecision in the texts of the 1990s, and addressing the causal relationship theorized between alarm, disorganization, and fear. The primary aim of the article will then be to clarify the relationship between the concepts of disorganized attachment and fear, and to dig beneath an undifferentiated concept of fear toward more precise characterizations of biologically channeled alarm. The value of this clarified account will be demonstrated through discussion of pathways to such behaviors, a topic which has often been obscured by conceptual imprecision regarding the fear–disorganization relationship. Our approach is influenced by Bowlby's (1973) guidance that with fear, “we are dealing, not with some single comprehensive form of behavior, but with a heterogeneous collection of interrelated forms, each elicited by a slightly different set of causal conditions and each having a distinctive outcome” (p. 114).

## FEAR WITHOUT SOLUTION

Main and Hesse (1990) began by stating the proposition that “disorganized/disoriented attachment behavior observed in the Ainsworth Strange Situation … suggests that the infant may at times be experiencing a fear or distress too intense to be deactivated through a shift in attention (the Ainsworth A pattern), yet at least momentarily cannot be ameliorated through approach to the attachment figure (the Ainsworth B and C patterns)” (p. 163), and “the fear the infant experiences stems from the parent as its source” (p. 182). In later texts, it is stated that whereas insensitive parenting will be associated with an organized insecure classification, “fear of the parents, in contrast, is expected to lead to disorganized attachment’ (Hesse & Main, 1999, p. 492). Such statements, at face value, readily imply to a reader that unassuaged fright (a) occurs in the moment during the Strange Situation; (b) is immediately experienced by the infant in relation to the caregiver; and (c) is causal in the production of the paradoxical injunction to approach and avoid the caregiver, understood to produce conflict at the level of behavior.

Many have read Main and Hesse as suggesting that a conflict between attachment and fear or, more narrowly, fear of a terrifying caregiver, is the *only* cause of behavior identified as indices of disorganized/disoriented attachment. It is common to encounter statements even by authorities in the field referencing Main and Hesse (1990) as saying that, for instance, “the essence of disorganized attachment is that the child is at times scared of the attachment figure” (van Rosmalen et al., 2014, p. 21). Such statements have fed the obviousness of the assumption that the experience of fear is the proximal, rather than a frequent historical, cause of disorganization. To take further recent examples, Paetzold et al. (2015, p. 147) stated that according to Main and Hesse, “infants in the disorganized category develop a fear of their attachment figures because these figures display frightening behaviors in their daily interactions with their children;” and Sinason (2016, p. 223) wrote that “Disorganized attachment refers to grossly disorganized behavior on the part of the infant or child: apprehension in the presence of the mother or primary caretaker.” Such a reification of the fear–disorganization relationship, for instance, also appeared in the recent proposals for National Institute for Health and Care Excellence (NICE) guidelines (2015) for clinicians in the Untied Kingdom wishing to assess and work with attachment‐related problems in children. Fortunately, these guidelines were largely corrected in the final version.

Hesse and Main (2006, pp. 310–311) acknowledged that how their earlier work was framed and argued appears to have misled readers. The 2006 article identified that the authors wish that they had made it clearer that they intended their emphasis on frightened or frightening caregiver behavior causing fear as “one highly specific and sufficient, but not necessary, pathway to D attachment status.” While they did point, for instance, to “confusion” as an alternative to “fear” as the proximal cause of disorganized attachment behavior in Main and Hesse (1990, p. 175), close examination of the 1990 text from the vantage of the present has suggested to us that the term *fear* was used in a way which was insufficiently specified. The 1990 chapter fell subject to a danger identified by Bowlby (1960) that “unfortunately in colloquial English the word ‘fear’ is used in many senses, often being synonymous with expectant anxiety and sometimes with fright” (p. 110).

A fundamental part of the field's difficulty in avoiding imprecise and extreme interpretations of Main and Hesse's hypothesis has been that the behaviors listed in the Main and Solomon (1990) indices *can* be boiled down to the experience of fear. Such interpretations are not simply wrong; however, interpreting all the different D behaviors as caused by fear depends on sliding uses of the concepts of “cause” and “fear,” leading to theoretical and explanatory muddle. As philosophers of science have emphasized, the idea of “cause” can actually be quite misleading if attention is not paid to the specific properties of the processes enacted and if distinctions are not drawn, for instance, between the kind of causes in question (Cartwright, 2014).

To offer an analogue, most things can be made to boil. This does not, however, invalidate the fact that different properties exist in different materials, leading to varying melting temperatures. Index VI (direct indices of fear of the caregiver) boils to the experience of fear rapidly, at a low temperature (i.e., at a low level of deductive reasoning), scaffolded by observation rather than requiring much in the way of inference. Someone viewing the recording of the Strange Situation can see the fear; it is manifest. An immediate conflict between attachment and the experience of fear suggests itself as the proximal mechanism, for instance, for the behavior listed in Index II by Main and Solomon (1990): “The infant displays marked fear simultaneously with proximity seeking or contact maintaining” (p. 136). By contrast, crying at the stranger's leavetaking or interrupting an approach with a bout of anger directed away from the parent (both Index III) have different properties—and have different “boiling points” for being understood as caused by fear. The reasoning required to understand these differing Index III behaviors as being caused by fear, if at all, is not the same. Yet, for both, the length of deductive reasoning required is too great to allow fear to be considered as the proximal “cause” in the way that it is possible with Index VI behavior. Moreover, it may quite reasonably be considered that a child whose attachment pattern has been disrupted by a short burst of anger is *proximally* experiencing anger, not fear.

Slade (2014) observed that in the context of familiar (if potentially quite unrepresentative) descriptions of disorganization as physical collapse to the floor and fleeing the caregiver in fright, “contemporary attachment theorists and researchers have been inclined, as the phenomena are so dramatic, to focus on the role of fear in disorganized attachment” (p. 256). Confusion between the ordinary meaning of the word “disorganization” (i.e., chaos) and the technical meaning in Main and Solomon (1990) (i.e., disruption of the attachment system) also has contributed to misunderstandings among some readers. Yet, the logic, if not some of the rhetoric, of Main and Hesse's position in the 1990s actually suggests that the child need not ever consciously experience the affect of fear for the paradoxical injunction towards and away from a caregiver associated with alarm to occur. This was highlighted in Hesse and Main (2006): “The activation, and perhaps at times perpetuation, of this particular form of attentional looping may well be associated with ‘prepared’ (evolutionarily channeled) fear, and hence, potentially occur outside of consciousness” (p. 310). That is, the approach–flight paradox may be so contrary to how evolution has wired the attachment system and its modes of perception and expectation that there is no reason to presume that all infants necessarily will consciously experience the fear derived from these circumstances.

A good deal of confusion about causality has stemmed from the fact that the term *fear* is used in four different ways in the Main and Hesse texts of the 1990s and, to an extent, still in Hesse and Main (2006) (see Table 1). Sometimes these senses overlap; however, more often they actually do not. A first use of the word “fear” in these texts is as a synonym for alarm, the biologically channeled psychophysiological response to a natural or learned cue for danger. A second sense of the word “fear” in these texts is as an immediate phenomenological experience in the mind of the infant. This is the sense most readily conjured by everyday language use of the word “fear” and so largely has dominated interpretations of the Main and Hesse texts. A third use of the term is as a synonym for “fright,” which otherwise has the technical meaning of alarm without assuagement in these texts. Fourth and finally, the word “fear” is used as a synonym for “apprehension,” which otherwise has the technical meaning of Index VI behavior. To illustrate these distinctions, note that Hesse and Main (1999) offered the suggestion that an infant's experience of chronic activation of the attachment system without assuagement will be associated with disorganized/disoriented attachment with an attachment figure in the Strange Situation. An example of such lack of assuagement would be a fortnight's separation, for instance, if the caregiver were hospitalized. Such chronic lack of assuagement of the attachment system can be understood to *alarm* the infant, and this alarm is in turn chronically unassuaged, which leads to *fright*. In this pathway, however, there is no anticipation that the infant will experience fear when he or she sees the caregiver, and an infant in such circumstances would not be chronically unable to find the caregiver as a safe haven and receive comfort—contrary to overgeneralizations of the idea of “fright without solution.” Furthermore, it can be added that if the separation were to continue for a substantial period, the child would be expected to move *from disorganization to reorganization*, likely to a relatively brittle avoidant strategy if he or she was to be reunited with that caregiver (see Bowlby, 1980; Crittenden, 1999; Solomon & George, 2016).

**Table 1:** Meanings of the Term Fear in Main and Hesse Texts of the 1990s

	Fear as *Alarm*	Fear as Immediate *Experience*	Fear as *Fright*	Fear as *Apprehension*
Description	The caregiver is associated with a natural or learned cue for danger for the infant.	The infant experiences fear in relation to the caregiver because he or she is perceived as a cue for danger.	The infant's alarm remains unassuaged because the caregiver cannot be accessed as a safe haven	The infant is scared of their caregiver. “Direct indices of apprehension,” in which the fear seems palpable rather than inferable, is one of the main and Solomon (1990) indices.
Location	Biologically channeled psychophysiological response	Phenomenological experience integrating expectation with present perception	The affect of unassuaged alarm	Observable behavior

Such analysis suggests a question largely neglected to date, as a result of reifications of the fear–disorganization relationship: *How or in what way is infant disorganized attachment behavior in the Strange Situation the result of fear?* The value of distinguishing this question will be demonstrated through consideration of three proposals for further pathways to infant disorganized attachment besides that suggested by Main and Hesse; these will be treated in chronological order. The first, Main and Stadtman (1981), has suggested a role for harsh rejection of the infant by the caregiver as a source of alarm; the second, by Lieberman and Amaya‐Jackson (2005), has emphasized trauma and emotional dysregulation; and the third, by Padrón et al. (2014), has proposed a temperamental pathway to disorganized infant attachment.

Our clarification of the Main and Hesse hypothesis allows a hitherto unrecognized pathway to disorganization to be discerned in the data presented by Main and Stadtman (1981), a text published before the introduction of the disorganized/disoriented classification. Clarification of the role of fear supports evaluation of discussions that reframe disorganized attachment as emotional or intersubjective dysregulation, such as Lieberman and Amaya‐Jackson (2005), which have been offered as an alternative paradigm to the Main and Hesse hypothesis. Finally, our respecification helps also to consider recent findings, reported by Padrón et al. (2014), which appear to distinguish among disorganized infants those with a temperamental vulnerability from those whose behavior more directly suggests fear of their caregiver.

## REJECTION AND FEAR

Main and Stadtman (1981) reported intriguing findings from three studies. In the first two studies, caregivers (*N* = 38 and *N* = 30, respectively) and their infants were invited by Main and Stadtman to participate in laboratory play sessions. Infants were observed interacting with the caregivers, and playing alone and with a stranger in the presence of the caregiver. The researchers were interested to examine the extent to which behavior from the caregiver which rebuffed the infant's attachment behaviors—which Ainsworth had found associated with the avoidant attachment pattern in the Strange Situation—was correlated with anomalous behaviors during free play:

A coder was instructed to tabulate any behavior that seemed odd or disturbing in itself (stereotypies, hand‐flapping, echoing) and any behavior that seemed odd or disturbing simply because it occurred out of context (e.g. when the toddler showed sudden inexplicable fear of a toy guitar, or laughed for no apparent reason). Although the coder was instructed only to tabulate “odd” behavior, a review of the behaviors actually tabulated showed that they could largely be described as conflict behaviors. (Main & Stadtman, 1981, p. 295)

Main and Stadtman found that these conflict behaviors had a .40 association with the degree to which infants were rejected by their caregiver when they made bids for closeness when it was just the two of them. This increased to a .63 association with caregiver rejection in the presence of a stranger. Main and Stadtman noted:

Many infants responded immediately with conflict behavior when rejected by the mother. For example, one infant was described as attempting several times to move away from the stranger and toward the mother when the stranger tried to engage her in interaction. The mother stiffened and pulled away from the infant in response, sometimes physically pushing the infant away. In apparent direct response to the mother's physical rejection the infant grimaced, engaged in odd and empty laughter, kicked her feet many times in sudden peculiar tension movements, and engaged in stereotypies. (p. 301)

Although occurring in the low‐stress condition of free play, and hence *not* an equivalent to disorganized attachment as a classification for the Strange Situation, this behavioral sequence appears to be the result of irresolvable conflict of intention between approach and avoidance. What is being assessed is “conflict behavior” rather than “disorganization.” Yet, conflict behavior comprises the first five of the seven indices of disorganized/disoriented attachment; the wide overlap between the two constructs means that this sequence manifestly meets the Main and Solomon (1990) criteria for behavioral disorganization.

To further interrogate the meaning of the correlation between caregiver rebuff and infant conflict behavior, in a third study, Main and Stadtman (1981) reanalyzed Ainsworth et al.’s (1978) narrative records of mother–infant interactions observed throughout the infant's first year. They found that the extent of the caregiver's aversion to contact had a .55 association with conflict behavior in the infant, and a .44 association with instances of overt aggression by the infant toward the caregiver. This is an important finding because caregiver aversion to contact at home was found to be almost ubiquitous among infants classified as avoidant in the Strange Situation (Ainsworth et al., 1978). Taken as a whole therefore, the findings from Main and Stadtman's studies appear to suggest that infants classified as avoidant in the Strange Situation are disproportionately likely to show behaviors listed in the Main and Solomon (1990) indices of disorganization, and aggression both at home and in free play with their caregiver.

Main and Stadtman (1981) theorized their results by considering that grossly aversive behavior by a caregiver in response to an infant's bids for contact may *itself* alarm an infant and provide him or her no opportunity for using the relationship to regulate distress. They reasoned that

because a frightened child inevitably seeks the attachment figure as a haven of safety, even the mother's own rejection of her infant draws the infant toward her. By repelling her infant, then, a mother simultaneously attracts him … this creates an irresolvable and ultimately self‐perpetuating conflict situation. (p. 293)

Main and Stadtman suggested that the infant experiences a conflict between distress and a desire for protection from the caregiver, and dispositions toward anger and withdrawal, which are not resolved within the relationship and will therefore be elicited whenever a situation normally arouses attachment behavior. They proposed, however, that one capacity that at least some infants can use to circumvent this conflict is physical and attentional avoidance, such as attending to the toys at the moment of reunion.

At a theoretical level, an infant showing an Ainsworth avoidant pattern is understood to experience a prohibition, encountered in the behavior of the caregiver, which inhibits expression or activation of his or her attachment system. He or she is therefore subject to a “conflict situation” when the attachment system would otherwise be activated. This situation is an alarming one for the infant in a sense, in that his or her anxiety is not resolved by the proximity and availability of the attachment figure. However, the hallmark of the avoidant infant is that he or she can shift attention away from the demands of the attachment system. This shift in attention resolves the potential conflict of intention between attachment and inhibition in a coordinated way. The infant adopting an avoidant strategy can receive a conditional proximity to a caregiver who is not a source of alarm; in this way, the infant is able to receive sufficient, indirect reassurance to allow self‐regulation and de‐escalation of the attachment system, although not comfort.

The position of this avoidant infant is in contrast to a conflict situation in which the caregiver is associated with alarm for the infant, as is more likely the case, for instance, if the parent displays manifestly frightened or frightening behavior. Hesse and Main (1999,) stated that “insensitivity to infant signals—whether displayed in persistent rejection of attachment behavior or in neglect, interference, or a failure to encourage the development of autonomy—is unlikely in itself to become alarming” (p. 498). Yet, Main and Stadtman (1981) represented an excluded middle: They described a situation in which the caregiver rebuffs the infant like the parent of an avoidant child, but in such a way that the aversive quality of the parental rejection itself elicits alarm in the infant, producing a paradoxical situation. The hypothesized pathway would be: aversive parental responses to child distress—alarm at the prospect of seeking the caregiver—some form of disorganized behavior in the Strange Situation. There will be some overlap, as Jacobvitz, Leon, and Hazen (2006) observed, between grossly aversive caregiver behaviors and alarming caregiver behaviors; however, the two are generally distinguishable. Unfortunately, in reifications of disorganization and fear, critical questions about different sources of alarm have been lost. As a result, despite the potential clinical relevance of this question, it remains largely unexplored whether a caregiver's rejection of a child when he or she seeks reassurance and comfort causes sufficient motivational conflict to produce an alarming conflict irresolvable by the infant and, as a result, an increase in disorganized attachment classifications among such infants in the Strange Situation.

There are, however, studies whose methods and findings offer evidence relevant to this concern. An unpublished paper by True, Lyon, Pisani, and Oumar (2003) specifically assessed aversive parental responses to infant approach behaviors in their Dogon sample from Africa. They found that parental aversion to contact with the infant explained a significant amount of the variance in infant disorganized attachment *over and above* parental frightening or frightened behavior. True et al. chose a culturally specific explanation for their findings, appealing to the particularity of Dogon caregiving practices in which breastfeeding is the most common response to infant distress, which means that “the Dogon infant of an aversive mother is, ultimately, caught in an approach‐avoidance conflict. He or she needs and wants to breastfeed so is motivated to approach. At the same time, the infant experiences close contact the mother as a source of tension so is motivated to avoid the person she or he wants to approach.”

True et al.’s (2003) findings support Main and Stadtman's (1981) hypothesis, but raise the question of whether grossly aversive responses to infant bids for contact also is a predictor of conflict behavior and/or disorganization in Western samples through the mechanism Main and Stadtman suggested. Evidence for the latter conclusion is that, as in the African sample of True et al., Moran Forbes, Evans, Tarabulsy, and Madigan (2008) found that parental insensitivity did predict infant disorganized attachment in a high‐risk Canadian sample. Moran et al. measured maternal sensitivity using Maternal Behavior Q‐sorts to assess behavior recorded during a 2‐hr home visit, a measure that would include grossly insensitive responses to infant bids for contact (also see Gedaly & Leerkes, 2016). Thus, while insensitivity in general is but weakly associated with the incidence of the infant disorganized attachment classification (van IJzendoorn, Schuengel, & Bakermans‐Kranenburg, 1999), grossly aversive caregiving in high‐risk samples may have a more significant role to play, as Main and Stadtman predicted.

Main and Stadtman's (1981) article also has potential significance for thinking about disorganized attachment and clinical and social welfare assessments. Social workers and clinicians have at times used observation of behavioral disorganization by an infant in the family home as sufficient reason to warrant scrutiny of the family by social services; Main, Hesse, and Hesse (2011, p. 441) observed with dismay that this practice is “widespread” in some areas, and it has received recent critical discussion from Granqvist et al. (2016). The social services assessment of disorganization in naturalistic settings such as the home appears to be based on the assumption that the behaviors specified by Main and Solomon (1990) in any setting are a stable “symptom” of fear of or for the caregiver. However, Main and Stadtman's findings have indicated that infants who hide their experience of conflict through display of an avoidant strategy in the Strange Situation may well show in the home setting precisely the behaviors in 1981 they were calling “conflict behaviors” and which largely overlap with the Main and Solomon indices. No study to date has examined this concern, although the home observation data are already available in some labs to assess differences in the naturalistic behavior of infants classified as organized‐avoidant and disorganized in the Strange Situation. Such observations offer important potential to help shed light on infants’ experiences of alarming caregiver, experiences that are assumed to be at the heart of disorganized attachment.

## TRAUMA AND EMOTIONAL DYSREGULATION

Over the past 10 years, the idea of a conflict between the attachment system and fear of an attachment figure as the mechanism of disorganized attachment has increasingly been construed instead as unmetabolized negative affect. Many researchers have shifted away from discussing disorganized attachment in terms of the “attachment system,” preferring instead discussion in phenomenological terms, appealing to the concept of “intersubjective dysregulation.” Although in subsequent developmental science articles (e.g., Bernier & Meins, 2008; DeOliveira et al., 2004; Wazana et al., 2015) appeal to intersubjective dysregulation is often shorn of psychoanalytic markers, consideration of citation histories has suggested that this language has its roots in papers by clinician‐researchers drawing on psychoanalytic theory (e.g., Cassidy & Mohr, 2001; Fonagy, Gyorgy, Jurist, & Target, 2003; Lyons‐Ruth, 1999, 2007). In these clinically oriented theoretical publications, disorganized attachment has often been understood on the model of Freud's (1995 [1920]) concept of trauma as a quantitative breach in the energetic barrier that organizes experience or through Bion's (1962) idea that infant experience needs to be contained and metabolized within the context of relationships or else becomes overwhelming to the child (e.g., see Fonagy et al., 2003, pp. 135–136). It is through this lens that the concept of disorganization is reinterpreted, grounded in clinical experience.

However, two points can be raised regarding theories of disorganization as intersubjective dysregulation and unmetabolized negative affect. First, other behaviors not coded as disorganized, such as ambivalent/resistant attachment, would not be discriminated from disorganization in the way that the idea of “dysregulation” is currently used. Neither Lyons‐Ruth's AMBIANCE nor Fonagy's measure of Reflective Functioning on the Adult Attachment Interview, measures focused on intersubjective dysregulation, discriminates the caregivers of infants classified as ambivalent/resistant from those classified as disorganized/disoriented in the Strange Situation (e.g., Kelly, Slade, & Grienenberger, 2005; Stacks et al., 2014). Second, not all of the Main and Solomon (1990) indices appear, at least phenotypically, to suggest dysregulation, although, of course, this depends entirely on what is intended by the term *dysregulation*. In using this term, there is a slippage in texts such as Fonagy et al. (2003) between the idea of the infant as (a) overwhelmed by intensity of distress, and (b) unable to manage distress in a behaviorally coherent way. Yet, there are clearly behaviors in the Main and Solomon indices—such as a depressed or an apathetic look during a coherently sequenced approach— not readily conceptualized as *either* an infant being overwhelmed by distress or unable to manage the distress. The concept of the failure of the interpersonal interpretive mechanism is surely a significant one, and its predictive validity can and should be tested, but it must be distinguished from disorganization as presently operationalized by the Main and Solomon coding system.

Although it may be gesturing to important causal processes, there has been little discussion of quality or severity of dysregulation, and in general, the concept of intersubjective dysregulation appears underspecified as an account of disorganized attachment. It can perhaps be usefully supplemented by renewed attention to the idea of *biologically channeled alarm* and the environmental conditions which may disrupt or produce contradiction for the attachment system. This can be shown by considering Lieberman and Amaya‐Jackson's (2005) discussion of the Main and Hesse hypothesis. Lieberman and Amaya‐Jackson proposed that a paradoxical injunction caused by fear of or for the caregiver is not the only pathway to infant disorganized attachment. Addressing the case of violence between a parent and his or her adult partner, Lieberman and Amaya‐Jackson proposed that infant disorganization can be expected whenever the parent fails as a protector in the context of ongoing traumatizing circumstances:

The inability to rely on the parent to restore self‐regulation, in turn, exacerbates the child's anxieties because the parent offers no reassurance that the child will not be abandoned, will continue to be loved, and will be protected from bodily harm … . This conceptualization highlights the simultaneous contributions of external trauma *and* maternal unavailability to the formation of disorganized attachment. This dual emphasis on external events and internal processes stands in contrast with the current emphasis on … maternal frightened/frightening behavior. (pp. 111–112)

Lieberman (2014) restated her proposal again more recently, in a reply to Slade's (2014) call for renewed critical attention to attachment–fear conflicts:

Dysregulated and traumatized parents can be very frightening to their children.… They transmit their internal disorganization to their children, not only by directing their anger, punitiveness, and unpredictability towards the child but also by exposing them to a cacophony of daily, real‐life situations that are helplessly witnessed or experienced by the child: domestic violence, fights within the family … long periods of not being aware of the child. (p. 278)

As proposed earlier, the logical core of the Main and Hesse hypothesis—although not its rhetorical thrust—was the idea that disorganized infant attachment in the Strange Situation would be predisposed by some situation, whether acute and traumatic at a particular historical moment or low‐level and structural over the span of the relationship, in which an attachment figure him‐ or herself was associated with alarm for an infant. This does not require the phenomenological experience of the caregiver as a source of fear. As such, we believe, the Lieberman and Amaya‐Jackson (2005) proposal is compatible rather than in competition with the Main and Hesse hypothesis. A cacophony of alarming experiences—combined with a caregiver's presence, but unavailability for protection and comfort—may lead that caregiver to be *associated* for the infant with alarm.

In agreement with Lieberman and Amaya‐Jackson (2005), in the coding manual to the Adult Attachment Interview, Main, Goldwyn, and Hesse (2002) specified that witnessing family violence could be potentially traumatic. Some other researchers also have found strong and chronic marital conflict associated with infant disorganized attachment (Owen & Cox, 1997; Solomon & George, 1999b; Zeanah et al., 1999). In our view, if a child hears banging and shouting and experiences alarm, and perceives *their safe haven is itself under attack*, then this can be expected to produce a paradoxical association between expectations of a safe haven and perceptions of threat which can then later shape the infant's expectations and behavior, in the Strange Situation. The child may well associate alarm with the very individual that the attachment system is prompting them to approach with the expectation of safety.

In the Strange Situation, the infant encountering a caregiver who, in his or her home life, has faced the kinds of predicaments Lieberman (2014) described will not only have fewer internal resources for achieving emotional or intersubjective regulation (as argued by Bernier & Meins, 2008) but also will be predisposed on seeing the attachment figure to recall the escalating alarm without assuagement (fright) or traumatizing events which occurred in his or her presence. Such associations may not readily be reconciled with the expectation of the caregiver as a safe haven. This conflict of expectations, provoking an approach–flight paradox, is likely to predispose at least some of the kinds of infant behaviors specified by Main and Solomon (1990). Lieberman's very valuable emphasis on a pathway to disorganized attachment through a cacophony of daily sources of alarm combined with caregiver emotional unavailability has found support in findings by Cyr et al. (2010). The meta‐analysis by Cyr et al. found that even where there was no known maltreatment, the ramifying intersection of multiple social and economic risks experienced by a family results in considerably elevated rates of infant disorganized attachment. Such a cacophony of difficulties may be anticipated to be associated with infant disorganization by *both* producing a frightening degree of chaos in the family system, and through increasing the parent's emotional unavailability to the infant and through making availability for repair more difficult, in the context of all these pressures.

## FEAR, PARENTING, AND TEMPERAMENT

Finally, a third proposed pathway to infant disorganized attachment also can be used to illustrate the value of increased precision around the relationship between fear and the behaviors listed in the Main and Solomon (1990) indicies. In a recent study, Padrón et al. (2014) examined whether infants from the Minnesota longitudinal study whose predominant form of disorganized behavior was from Index VI (apprehension) or VII (disorientation) differed from other infants coded as “D.” This developed the work of Spangler (2011), who has particularly argued that some disorganization may have a temperamental contribution rather than being entirely the product of the history of experiences with the caregiver. Padrón et al. found that infants whose predominant form of disorganization was from Index VI or VII did not have difficulties with emotion regulation in their first 2 weeks of life, and had parents who were observed to score poorly on the Ainsworth Sensitivity/Insensitivity and Cooperation/Interference scales in the home. In contrast, those infants whose predominant form of disorganized behavior was from Indices I to V were found to be significantly more likely to have had difficulties with emotion regulation on the Brazelton Noenatal Behavioral Assessment Scale already in their first 2 weeks of life. Padrón et al. (2014) interpreted their findings with the proposal that “the direct indices of fear and apprehension displayed by infants in the Frightened group were more likely to have emerged in response to exposure to frightening caregiving behavior from the external environment. On the other hand, it was thought that infants who primarily displayed other disorganized behaviors, in the absence of fear, might have been more likely to have had compromised internal regulatory abilities at birth” (p. 206). These researchers argued that their findings suggest that temperament represents an alternative pathway to certain forms of disorganized behaviors as seen in the Strange Situation.

This study is a groundbreaking and welcome contribution to thinking about disorganized attachment. We would raise two questions in reflecting on its conclusions. First, Padrón et al. (2014) distinguished Index VI and VII behaviors because these two classes “are indicative of direct expressions of fear or disorganization in response to the parent: direct indices of apprehension regarding the parent, and direct indices of disorganization or disorientation (Main & Solomon, 1990).” Therefore, Padron and colleagues argue, it is possible to distinguish among disorganized infants on the basis of whether they directly exhibit apprehension in the Strange Situation” (p. 202). This latter statement does not follow, however, from the first. Index VI behaviors are exhibitions of apprehension in the Strange Situation. However, Index VII behaviors suggest confusion or disorientation rather than “direct expression of fear. There is no argument given by Padrón et al. as to why Index VII is more clearly an exhibition of fear or apprehension than, for instance, freezing on reunion with the parent, Index V. Surely Padrón et al. placed VI (direct indices of apprehension) together with VII (disorientation) as a single group since both can be expected to be especially associated with risk. To these considerations, we can add that the “direct indices of apprehension” (Index VI) in Main and Solomon (1990) indices were disproportionately common from the tapes examined by Main and Solomon from high‐risk and maltreating samples, rather than the tapes from low‐risk samples. Yet, further research would need to formally establish this.

Second, Padrón et al. (2014) concluded that frightening caregiving behavior causes the fear and apprehension displayed by the infants in the Frightened group. Brazelton scores do distinguish infants whose predominant form of disorganized attachment falls in Indices I to V, as compared to those where the predominant behaviors are VI and VII, *F*(1, 35) = 9.190, *p* < .01. This is a highly notable finding. However, Padrón et al. did not report data that suggest that caregiving behavior significantly distinguishes these groups of “D” infants: Both groups have comparatively insensitive caregivers. Furthermore, there is no evidence provided that either group has caregivers eliciting *alarm*. These researchers do not have available an assessment of frightening caregiving behavior (p. 207). Instead, they reported on caregiving behavior using the Ainsworth scales for parental Sensitivity and Cooperation/Interference. Based on the idea that high levels of interference will be experienced by the infant as incomprehensible, Padrón et al. suggested that “maternal interfering behaviors may contribute to the development of disorganization” (p. 207). Drawing on the distinctions from Table 1, it can be observed that while interfering behaviors may be incomprehensible for the infant even to the point of generating fear, it is not clear that interfering behaviors make the caregiver a source of *alarm* for the infant, per se, although Main and Stadtman's (1981) results have suggested that interference may increase the likelihood of a disorganized classification in the Strange Situation when its intensity or manner alarms the child.

## PATHWAYS TO DISORGANIZATION

The primary aim of this article was to clarify the relationship between fear and disorganization. However, in closing, we wish to offer a few further testable speculations, which stem from clarification of the relationship between concepts of disorganization and fear. Already a decade ago, Hesse and Main urged that that it would be ‘a worthwhile endeavor’ to consider ‘the forms of D behavior exhibited by their infants’ and reflect on potential differences in their origins (Hesse and Main 2006: 335). We repeat this call here, but add a word of caution. Such distinctions need to be ballasted by overarching classifications to support cumulative research agendas. As Pianta, Egeland, and Sroufe (1990) argued, aggregation of related phenomena has significant advantages for prediction and statistical power, and should be a mainstay of scientific inquiry. However, Pianta et al. advised that it also is “relevant and researchable” (p. 230) to examine the relative importance of the elements and their interrelations within a construct, as well as whether some elements predict outcomes of interest better or differently (e.g., see Abrams, Rifkin, & Hesse, 2006).

Something that Main and Hesse's work of the 1990s has in common with the three texts considered in the previous sections is that all of them have suggested causal pathways for particular forms of behavioral disorganization more than have others. These pathways have often, but not always (e.g., Waters & Crowell, 1999), remained out of view in part due to a lack of clarity regarding the concept of fear. The behaviors listed by Main and Solomon (1990) are likely not evenly distributed among these different pathways, an important point for future research and one largely invisible to the field in our view.

Main and Hesse (1992 1992) suggested that parental frightening, frightened, and dissociative behavior will be associated with the infant disorganized classification. However, they also hypothesized that dissociative caregiver behavior is likely to be discriminatively associated with the behaviors suggestive of dissociation or disorientation among the Main and Solomon (1990) indices. This claim was then repeated in Main and Morgan (1996), and then in Hesse and Main (2006), who distinguished between manifestly apprehensive behaviors and those that appear more overtly dissociative: “While many D behaviors identified as disorganized are unlikely dissociative, as hiding under the chair at the entrance of a clearly frightening mother, some D behaviors (chiefly trance‐like behaviors and seemingly dissociated actions) do seem to fit a dissociative model” (p. 334).

Main and Stadtman (1981) suggested that grossly aversive caregiving may leave infants inclined to attempt an avoidant strategy, but too distressed to do so in a coherent manner. The distress may bleed through the avoidance, leading to contradictory forms of behavior. Grossly aversive behaviors by a caregiver could be predicted to be disproportionately associated with approach–avoidance conflicts (Indices I–III) by their infant in the Strange Situation. Again, this would need to be established by research.

Fonagy et al. (2003) situated affective dysregulation as the fundamental process of disorganized attachment. Yet, note that not all behaviors in the Main and Solomon (1990) indices appear affectively dysregulated, and vice versa. For instance, approaching the caregiver with a depressed look is not obviously the same thing as being flooded by distress. Distress can be inferred from a depressed expression on reunion, but there has been a shift in the level of analysis, with the concept of “dysregulation” sliding between different usages. Crying while turned away from the caregiver in Index III seems a paradigmatic example of dysregulation. Further discussion is needed about how such behaviors relate to the ideas of fear and alarm, and perhaps comparison with other kinds of behavior in the Main and Solomon indices such as stereotypies and direct indices of apprehension.

Padrón et al. (2014) reported that Brazelton scores in the first 2 weeks of life distinguish infants classified as disorganized, but who predominantly show behavior in Indices I to V rather than direct indices of apprehension, disorganization, or disorientation. Research is ongoing in Gottfried Spangler's lab to further explore the possibility that temperament may play a larger role for some forms of disorganization than for others. Such research offers the prospect of helping the field understand more about different forms and degrees of fear, and differences in the behavioral profile which may result.

Finally, Granqvist et al. (2016) raised the prospect that some behaviors in Index IV may represent less disorganization or dysregulation, per se, and instead are behaviors characteristic of neurodevelopmental disorders. Stereotypies were already recognized by Main and Solomon (1990) as weak indices of disorganization. They were included as a disorganization index because they seemed to represent conflict about displaying anger or distress when approaching the parent or when in the parent's arms. However, none are italicized markers, as it was known that stereotypies can reflect a variety of sources of stress or conflict (Sroufe, Stuecher, & Stutzer 1973). Their use for coding disorganization has subsequently been challenged because stereotypies are also characteristic of disorders on the autism spectrum (Willemsen‐Swinkels, Bakermans‐Kranenburg, Buitelaar, van IJzendoorn, & van Engeland, 2000). The question of the discriminant validity of stereotypies as indices of disorganization is therefore an interesting one for future research in deepening our understanding of the relationship between behavior and disorganization of the attachment system. However, it is not a question with significant implications to the present coding system, which already downplays stereotypies.

### Conclusion

We have presented here a reconsideration of the Main and Hesse hypothesis, clarifying the theoretical links posited among fear, disorganization, and alarm. We have used the conceptual distinctions drawn to consider three proposed alternate pathways to disorganization: grossly aversive rejection (Main & Stadtman, 1981), traumatic events and emotional dysregulation (Lieberman & Amaya‐Jackson, 2005), and infant temperament (Padrón et al., 2014; Spangler, 2011). All three proposed pathways are of interest, and important aspects of each account and how to test them may be obscured if the fear–disorganization relationship is not dealt with in a conceptually precise way. Conceptual precision has to date been reduced by formulations within Main and Hesse's early texts and also how they have been read.

Great caution is needed, especially from practitioners, since we know terribly little at this stage about the relationship between different pathways and behaviors. Yet, these reflections may still be relevant to practitioners in the context of formulation when thinking about situations where a caregiver is a potential source of alarm for their child. In pursuing these questions, and in avoiding the misapplications of the concept detailed by Granqvist et al. (2016), we anticipate that renewed discussion of fear and disorganization will be needed, and have worked here to help clear the path for future rigorous thinking and empirical research.
